# Hematological Reference Intervals for Healthy Iranian Blood Donors

**Published:** 2017-10-01

**Authors:** Mahboubeh Rasouli, Mojgan Pourmokhtar, Shaghayegh Sarkardeh

**Affiliations:** 1 Department of Biostatics, School of Public Health, Iran University of Medical Sciences, Tehran, Iran; 2 Blood Transfusion Research Center, High Institute for Research and Education in Transfusion Medicine, Tehran, Iran; 3 Faculty of Pharmacy, Pharmaceutical Sciences Branch, Islamic Azad University, Tehran, Iran

**Keywords:** Reference values, Blood donors, Iran, Gender, Age

## Abstract

**Background:** Development of locally-derived hematological reference intervals is necessary for improving the quality of health care and clinical trials. However hematological reference intervals are affected by several variables including age, gender and environmental factors. Therefore this study was conducted to determine the gender and age-specific hematological reference intervals of healthy Iranian blood donors.

**Materials and Methods: **Selected hematological indices of 394 healthy blood donor volunteers, donating blood at Tehran Blood Transfusion Center were analyzed. Hematological reference intervals, stratified by age and gender were compared. The results of current study were also compared with those of US population.

**Results:** There were significant gender-related differences for mean values of hematological indices, with males having higher mean values of RBC, HCG, HCT and MCV than females. While the mean of PLT and MCH were higher in women. Age-related differences for mean values of RBC and MCH were also significant. The comparison of reference intervals, stratified by both gender and age showed that RBC, HGB and HCT values were higher in males than females in all age groups. But MCH values of females in all age groups and WBC and PLT counts in females older than 30 years were higher compared to the males in the same age group. The results of this study showed some similarity with US population, with narrower intervals.

**Conclusion: **This study suggests that gender and age-specific, locally derived hematological reference intervals should be referred to, before interpretation of any laboratory test result.

## Introduction

 The emersion of automatic cell counting and data processing has made it feasible to examine and evaluate a larger amount of data with relatively higher degree of precision. Nevertheless, for effective interpretation and distinction, it is necessary to establish accurate reference values, or more precisely, the interval of reference values^1^^,^^2^. The concept of reference value, launched by Saris and Grasbeck in 1969, can be expressed as the establishment and use of relevant data for interpreting laboratory tests and medical observations^3^. In daily practice, reference intervals are necessary for health assessment, diagnosis orientation, treatment decision and patient management^4^^,^^5^. They are also important in Clinical trials for appropriate screening of volunteers, monitoring disease progression, and evaluating possible toxicity and adverse events^5^^,^^6^. Therefore, identifying the reference intervals is not only an important aspect of laboratory medicine but also will in turn reduces the treatment cost^2^.

Hematological reference values which are currently used in Iran have been adopted from textbooks or guidelines, referring mainly to European or American populations. But in fact hematological reference intervals are affected by several variables, including age ^7^ , gender ^8^, ethnicity ^9^, geography, pathogens^10^ and environmental factors such as climate, and altitude^7^. Besides, according to a number of related studies, some discrepancies in hematological reference intervals have been observed between Asian and African populations compared to European and American countries^2^^,^^8^^,^^10^^-^^19^. Therefore, the development of locally-derived hematological reference intervals may be beneficial for effective interpretation and distinction and subsequently for improving the quality of health care. Moreover, it prevents unnecessary follow-up investigations, treatment and mismanagement of patients and also excludes selection bias and misclassification of adverse events in clinical trials. Therefore, this study was conducted to determine the hematological reference intervals of healthy blood donors in Tehran Blood Transfusion Center as an example of the Iranian population. 

## MATERIALS AND METHODS

**Subjects**

A total number of 394 subjects (327 males and 67 females) whose age ranged between 19-64 years were randomly selected from a population of healthy adult blood donor volunteers donating blood at Tehran Blood Transfusion Center (TBTC) between Nov. 2014 to Oct. 2015. The inclusion criteria were good health condition of donors based on medical history, physical examination and seronegative laboratory findings for human immunodeficiency virus antibody test (anti-HIV), Hepatitis B surface antigen test (HBsAg), hepatitis C antibody test (anti-HCV) and rapid plasma reagin test for Syphilis (RPR). 

**Sample collection**

Blood samples were drawn from each subject by antecubital venipuncture using vacutainer tubes containing Ethylenediaminetetraacetic acid (EDTA) as an anticoagulant. All EDTA blood samples were analyzed within 24 hours of collection. 

**Serological testing**

All samples were tested for anti-HIV, HBsAg and anti-HCV by enzyme-linked immunosorbent assay (ELISA) using commercially approved test kits. Samples were also tested for syphilis, and those found to be positive in any of above tests were excluded from the data set.

**Hematological analysis**

Hematological analysis, including a complete blood count (CBC), was performed on EDTA anticoagulated blood samples using the automated hematology analyzer (Sysmex KX-21N, Japan). The hematological indices analyzed were hemoglobin (HGB), hematocrit (HTC), erythrocyte count (RBC), mean corpuscular volume (MCV), mean corpuscular hemoglobin (MCH), mean corpuscular hemoglobin concentration (MCHC), leukocyte count (WBC) and platelet count (PLT).

**Ethical consideration**

The study received ethical approval from the Ethics Committee of the Islamic Azad University and informed consent was obtained from all participants. A confidential pre-donation evaluation was conducted by a trained consultant physician to determine eligibility to donate blood. Confidentiality was assured by assigning anonymous identification codes to each participant. 

**Statistical analysis**

Data were entered into a Microsoft Excel database (Microsoft Corp., Redmond, WA, USA) and were statistically analyzed using the statistical package for social science (SPSS), version 23 (SPSS Inc., Chicago, IL, USA). Descriptive statistics including means, medians and their standard deviation (SD) with 95% confidence intervals (CI) and 95th percentile ranges were calculated for selected hematological parameters. The reference range intervals were calculated as the range between those values at the 2.5% and 97.5% limits for the population. Separate reference intervals for subclass age and gender were calculated. Age-and gender-stratified reference intervals were also determined. Student’s t-test and Mann-Whitney test were used to compare hematological parameters between genders. ANOVA and Kruskal-Wallis Tests were used to assess the relationship between hematological parameters and age. P-value less than 0.05 was considered as statistically significant. The results from the standards currently used for the US population were compared with those of our study based on the 95% confidence interval for the mean obtained in the present study. The mean of the specific test was considered to be significantly different from our study if its value was not included in the 95% confidence interval in our study.

## Results

 Among the 394 blood donor volunteers, 327 were males (83%) and 67 were females (17%). The mean values with 95% confidence intervals and medians with 95th percentile ranges for selected hematological parameters of Iranian blood donors, stratified by sex, are presented in Table 1. Males had higher mean RBC (5.62 × 10^12^/l versus 5.01 × 10^12^/l), HCG (16.03 g/dl versus 14.63 g/dl), HCT (48.33% versus 44.90%), MCV (85.03 fl versus 84.60 fl) and MCHC (32.80 g/dl versus 32.68 g/ dl) than females, while the mean of WBC (7.18 × 10^9^/l versus 7.23 × 10^9^/ l), PLT (228.79× 10^9^/l versus 255.03× 10^9^/l ) and MCH (28.53 pg versus 29.26 pg) were higher in women. According to Student’s t-test and Mann-Whitney test, there were significant gender-related differences for mean values of RBC, HCG, HCT, MCV, MCH and PLT (P<0.05), but no statistically significant gender differences were observed for mean values of WBC (P=0.800) and MCHC (P=0.151). 

Reference intervals were calculated for 4 age groups including less than thirty, 30-40, 41-50 and more than 50 years. Table 2 lists the mean values with 95% confidence intervals and medians with 95 percentile ranges of selected hematological parameters stratified by age. Variance analysis showed significant age-related differences in mean values of RBC (P<0.001) and MCH (P=0.005). These significant differences in mean values of RBC were observed between age groups of 30-40 and >40 (5.65 × 10^12^/l versus 5.41 × 10^12^/l), while significant differences in values of MCH were observed between age groups of <30 and 41-50 (28.26 pg versus 29.04 pg). 

**Table 1:** Mean values with 95% Confidence Intervals and medians with 95 percentile ranges of hematological parameters of Iranian blood donors

**Sex**	**Female**		**Male**		**Total**	
**Parameter**	**Median (95** **Percentile Range)**	**Mean (95% CI)**	**Median (95** **Percentile Range)**	**Mean (95% CI)**	**Median (95** **Percentile Range)**	**Mean (95% CI)**
RBC (× 10^9^/ l)	4.30 (4.92-5.90)	5.01 (4.92-5.09)	5.64 (4.72-6.59)	5.62 (5.57-5.67)	5.54(4.58-6.41)	5.52 (5.47-5.57)
HGB (g/dl)	14.50(11.67-17.26)	14.63(14.36-14.90)	16.10(13.42-18.40)	16.03(15.88-16.18)	15.85(12.99-18.40)	15.79(15.65-15.94)
HCT (%)	44.40(37.27-54.59)	44.90(43.94-45.86)	48.40(39.92-55.50)	48.33(47.90-48.76)	47.90(38.61-55.50)	47.74(47.33-48.15)
MCV (fI)	88.60(84.89-101.03)	84.60(77.19-92.00)	85.60(77.52-96.50)	85.03(83.52-86.55)	86.15(76.89-96.75)	84.96(83.20-86.72)
MCH (pg)	29.50(24.40-32.23)	29.26(28.81-29.70)	28.50(24.94-31.40)	28.53(28.35-28.71)	28.70(24.89-31.72)	28.65(28.49-28.82)
MCHC (g/dl)	32.90(24.64-36.05)	32.68(32.19-33.17)	33.30(30.16-35.68)	32.80(32.25-33.36)	33.30(28.90-35.71)	32.78(32.31-33.25)
WBC (× 10^9^/ l)	7.10(4.07-11.45)	7.23(6.80-7.66)	7.00(4.42-10.70)	7.18(7.00-7.35)	7.00(4.39-10.70)	7.18(7.03-7.34)
PLT (× 10^9^/ l)	261.00(84.90-403.80)	255.03.(238.27- 271.79)	230.00(131.80- 351.80)	228.79(222.62- 234.95)	233.00(125.12- 361.88)	233.25(227.34- 239.15)

RBC: red blood cell; HGB: hemoglobin; HCT: hematocrit; MCV: mean corpuscular volume MCH: mean corpuscular hemoglobin; MCHC: mean corpuscular hemoglobin concentration; WBC: White blood cells; PLT: platelets

**Table 2:** Mean values with 95% confidence Intervals and medians with 95 percentile ranges of selected hematological parameters of Iranian blood donors (Stratified by age)

**Age group**	**<30** **(yrs.)**		**30-40** **(yrs.)**		**41-50** **(yrs.)**		**>50** **(yrs.)**	
**Parameter**	**Mean** **(95%CI)**	**Median** **(95percentile** **range)**	**Mean** **(95%CI)**	**Median** **(95percentile** **range)**	**Mean** **(95%CI)**	**Median** **(95percentile** **range)**	**Mean** **(95%CI)**	**Median** **(95percentile** **range)**
RBC (× 10^9^/ l)	5.59 (4.45-5.73)	5.65 (4.59-6.81)	5.65 (5.57-5.72)	5.72 (4.66-6.39)	5.41 (5.32-5.50)	5.43 (4.26-6.22)	5.41 (5.32-5.50)	5.42 (4.57-6.14)
HGB (g/dl)	15.79 (15.37-16.20)	15.90 (10.98-19.36)	16.07 (15.84-16.30)	16.10 (13.69-18.31)	15.70 (15.48-15.92)	15.65 (13.31-18.11)	15.62 (15.33-15.91)	15.85 (12.94-18.62)
HCT (%)	47.85 (46.68-49.03)	48.40 (36.58-57.51)	48.44 (47.82-49.06)	48.60 (41.16-54.37)	47.30 (46.56-48.04)	47.00 (37.87-55.12)	47.36 (46.53-48.18)	47.55 (38.61-54.90)
MCV (fI)	83.78 (79.80-87.76)	86.00 (75.40-96.41)	84.46 (81.42-87.50)	85.30 (77.24-96.55)	86.09 (82.91-89.26)	86.60 (79.59-97.97)	85.36 (80.74-89.98)	87.30 (56.29-97.12)
MCH (pg)	28.26 (27.88-28.63)	28.30 (24.54-31.57)	28.50 (28.19-28.80)	28.55 (25.09-31.72)	29.04 (28.74-29.34)	29.10 (25.27-32.15)	28.89 (28.51-29.28)	28.90 (25.17-32.11)
MCHC (g/dl)	32.31 (30.84-33.78)	33.30 (26.71-36.15)	33.17 (32.95-33.39)	33.20 (30.70-35.51)	33.20 (32.89-33.51)	33.50 (28.30-36.11)	32.22 (30.55-33.89)	33.00 (25.09-36.42)
WBC (× 10^9^/ l)	6.99 (6.60-7.38)	6.75 (3.77-10.88)	7.41 (7.12-7.69)	7.45 (4.79-10.70)	7.12 (6.84-7.41)	6.90 (4.89-10.81)	7.14 (6.79-7.50)	7.15 (4.10-11.34)
PLT (× 10^9^/ l)	228.10 (215.17-241.04)	234.50 (70.28-360-55)	232.66 (221.21-244.11)	233.00 (95.48-343.75)	241.13 (230.40-251.85)	238.50 (129.83-402.60)	226.37 (213.12-239.62)	213.50 (140.33- 383.75)

RBC: red blood cell; HGB: hemoglobin; HCT: hematocrit; MCV: mean corpuscular volume MCH: mean corpuscular hemoglobin; MCHC: mean corpuscular hemoglobin concentration; WBC: White blood cells; PLT: platelets

**Figure1:**
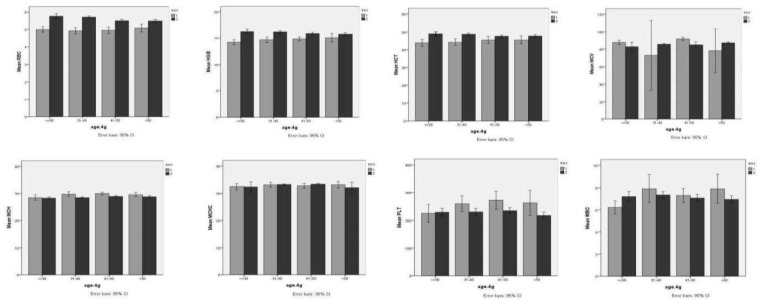
Variations in profile of hematological parameters of Iranian blood donors according to age and sex (1=Female, 2= Male)

The mean values with 95% confidence intervals and medians with 95 percentile ranges for selected hematological parameters stratified by both age and sex are presented in Table 3. According to Kruskal-Wallis Tests, there were significant age-related differences in the mean values of RBC (P<0.001), HGB (P=0.007), HCT (P=0.005), MCV (P=0.002) and MCH (P=0.043) among males. In other words, in males, the distribution of RBC, HGB and HCT mean values almost showed decline with aging, while positive correlation was almost found among age groups and both MCV and MCH mean values. As shown in Table3, the lowest mean of MCV (82.64 fl) and MCH (28.22 pg) in males were seen in <30 age group, while the highest mean of MCV (87.11 fl) and MCH (28.84 pg) were found in age groups of >50 and 41-50, respectively. Besides, it can be concluded that mean values of platelet count increased until the age of 50, but then declined in both genders. Moreover, according to variance analysis, there were also significant age-related differences in the WBC mean values (P=0.014) among females, and these differences were observed between age groups of <30 and 41-50 (6.23 × 10^9^/l versus 7.32 × 10^9^/ l).Variations in the profile of hematological parameters of Iranian blood donors based on the age and sex are shown in Figure1.

According to this Figure, there are some differences in RBC, HGB and HCT values by sex, indicating that males have higher values than females in all age groups (Table 2). By contrast, MCH values of females in all age groups and WBC and PLT counts in females older than 30 years were higher compared to the males in the same age group.

**Table 3:** Mean values with 95% confidence intervals and medians with 95 percentile ranges of selected hematological parameters of Iranian blood donors (Stratified by age and Sex)

**Age group**		**<30** **(yrs.)**		**30-40** **(yrs.)**		**41-50** **(yrs.)**		**>50** **(yrs.)**	
**Parameter**	**Sex**	**Mean** **(95%CI)**	**Median** **(95percentile** **range)**	**Mean** **(95%CI)**	**Median** **(95percentile** **range)**	**Mean** **(95%CI)**	**Median** **(95percentile** **range)**	**Mean** **(95%CI)**	**Median** **(95percentile** **range)**
RBC (× 10^9^/ l)	M	5.76 (5.61-5.92)	5.80 (4.05-6.86)	5.76 (4.82-6.39)	16.20 (15.97-16.43)	5.50 (5.42-5.59)	5.54 (4.34-6.33)	5.49 (5.40-5.58)	5.43 (4.76-6.22)
	F	5.01 (4.85-5.16)	4.93 (4.58-6.00)	4.90 (4.61-5.35)	14.66 (14.11-15.21)	4.96 (4.78-5.15)	4.91 (4.08-5.43)	5.09 (4.87-5.30)	5.00 (4.39-5.76)
HGB (g/dl)	M	16.26 (15.79-16.73)	16.50 (9.62-19.87)	16.20 (15.97-16.43)	16.35 (13.74-18.33)	15.88 (15.64-16.12)	15.80 (1349-18.35)	15.76 (15.45-16.06)	15.90 (12.38-17.89)
	F	14.18 (13.68-14.68)	14.30 (11.60-15.50)	14.66 (14.11-15.21)	14.40 (13.70-15.99)	14.86 (14.47-15.25)	14.90 (13.00-16.10)	15.07 (14.22-15.91)	14.50 (13.10-17.48)
HCT (%)	M	49.02 (47.72-50.32)	49.30 (32.14-57.74)	48.82 (48.21-49.43)	48.80 (41.84-54.59	47.66 (46.88-48.45)	47.50 (38.15-54.90)	47.80 (46.95-48.65)	47.70 (40.03-56.89)
	F	43.89 (41.88-45.90)	43.25 (37.20-53.49)	44.41 (42.57-46.25)	44.25 (40.70-48.54)	45.58 (43.56-47.60)	45.60 (38.00-50.70)	45.56 (43.21-47.91)	44.40 (38.70-53-10)
MCV (fI)	M	82.64 (77.54-87.73)	84.70 (30.98-94.47)	85.54 (84.71-86.37)	85.00 (77.81-96.73)	84.88 (81.10-88.67)	85.90 (77.88-97.72)	87.11 (86.00-88.23)	86.80 (74.41-96.85)
	F	87.66 (84.89-90.43)	87.85 (76.90- 101.51)	72.96 (32.99- 112.93)	90.00 (85.80-95.69)	91.85 (89.90-93.80)	91.90 (86.10-96.70)	78.23 (53.24- 103.23)	88.60 (84.50-96.20)
MCH (pg)	M	28.22 (27.82-28.62)	28.20 (24.14-31.27)	28.38 (28.06-28.69)	28.50 (25.04-31.50)	28.84 (28.50-29.17)	29.00 (24.37-32.55)	28.72 (29.29-29.16)	28.80 (23.04-31.45)
	F	28.38 (27.38-29.38)	28.85 (24.70-32.22)	29.77 (28.84-30.70)	29.65 (27.60-31.84)	30.00 (29.45-30.55)	30.10 (28.40-31.90)	29.59 (28.75-30.42)	29.50 (27.30-32.14)
MCHC (g/dl)	M	32.27 (30.38-34.16)	33.35 (12.70-36.84)	33.18 (32.95-33.41)	33.10 (30.70-35.53)	33.30 (32.96-33.63)	33.60 (28.84-36.35)	32.00 (29.93-34.07)	33.00 (3.54-35.94)
	F	32.45 (31.36-33.53)	33.10 (26.10-35.29)	33.05 (32.14-33.96)	33.30 (30.90-34.58)	32.74 (31.87-33.62)	32.90 (28.30-35.00)	33.12 (31.92-34.32)	33.00 (29.50-36.10)
WBC (× 10^9^/ l)	M	7.21 (6.75-7.67)	7.10 (3.59-11.67)	7.36 (7.06-7.65)	7.40 (4.77-10.63)	7.08 (6.76-7.40)	6.90 (4.65-11.05)	6.96 (6.63-7.28)	7.10 (4.27-9.45)
	F	6.23 (5.63-6.83)	6.00 (4.00-8.30)	7.91 (6.66-9.16)	7.80 (5.70-10.67)	7.32 (6.69-7.94)	7.20 (5.00-9.30)	7.91 (6.62-9.19)	7.80 (4.10-11.50)
PLT (× 10^9^/ l)	M	229.00 (214.67- 243.33)	234.50 (76.20- 364.23)	230.13 (217.89- 242.37)	231.00 (93.73-346-25)	234.59 (223.75- 245.44)	233.00 (137.70- 348.50)	217.41 (205.38- 229.44)	212.00 (142.65- 347.10)
	F	225.05 (192.99-257- 11)	230.00 (66.00- 358.20)	259.50 (231.53- 287.47)	268.50 (196.00- 313.90)	272.42 (239.38- 305.46)	263.00 (112.00- 399.00)	262.80 (217.22- 308.38)	254.00 (132.00- 387.00)

RBC: red blood cell; HGB: hemoglobin; HCT: hematocrit; MCV: mean corpuscular volume MCH: mean corpuscular hemoglobin; MCHC: mean corpuscular hemoglobin concentration; WBC: White blood cells; PLT: platelets

The comparison between hematological reference intervals obtained in the current study and US population is presented in Table 4. By comparing these values, it was found that the lower limits of the hematological ranges obtained in the current study were higher than those derived from the US population. Besides, the differences between the upper and lower limits of hematological reference values were much less in this study than in the US population.

**Table 4:** Comparison of hematological Reference Intervals obtained in current study with US Population

**Parameter**	**Current study**			**US population Range**
	**Female**	**Male**	**Total**	
RBC (× 10^9^/ l)	4.92-5.09	5.57-5.67	5.47-5.57	F:3.8-5.1 M:4.3-5.7
HGB (g/dl)	14.36-14.90	15.88-16.18	15.65-15.94	F:12.0-16.0 M:13.5-17.5
HCT (%)	43.94-45.86	47.90-48.76	43.33-48.15	F:35-45 M:39.49
MCV (fI)	77.19-92.00	83.52-86.55	83.20-86.72	80-100
MCH (pg)	28.81-29.70	28.35-28.71	28.49-28.82	26-34
MCHC (g/dl)	32.19-33.17	32.25-33.36	32.31-33.25	31-37
WBC (× 10^9^/ l)	6.80-7.66	7.00-7.35	7.03-7.34	4.5-11
PLT (× 10^9^/ l)	238.27-271.79	222.62-234.95	227.34-239.15	450-150

RBC: red blood cell; HGB: hemoglobin; HCT: hematocrit; MCV: mean corpuscular volume MCH: mean corpuscular hemoglobin; MCHC: mean corpuscular hemoglobin concentration; WBC: White blood cells; PLT: platelets

## Discussion

 The development of locally-derived hematological reference intervals may be beneficial not only for improving the quality of health care by making appropriate interpretation of the medical observations and laboratory tests results but also for the design, conduct and evaluation of clinical trials for biomedical interventions. Since hematological reference intervals currently used in Iran have been adopted from textbooks or guidelines referring mainly to European or American populations and according to some discrepancies in hematological reference intervals observed between Asian populations compared to European and American countries^2^^,^^20^, this study was conducted to determine the hematological reference intervals of healthy blood donors who referred to Tehran Blood Transfusion Center. Tehran was chosen because it is the largest city in Iran in terms of industry and economy and its inhabitants have migrated from different cities of Iran. Therefore, its population can be considered as a representative of the Iranian population. However, knowing that the hematological reference values can be affected by age and gender^7^^,^^8^^,^^16^, in this study, separate reference intervals were calculated for age and gender subclasses. The results indicated that there were significant gender-related differences for mean values of RBC, HGB, HCT, MCV, MCH and PLT, but no statistically significant gender-related differences were observed for mean values of WBC and MCHC. Meanwhile, higher values of RBC, HGB and HCT and lower values of PLT and WBC in males, compared with women, were similar to the findings of other previous studies^2^^,^^10^^-^^11^^,^^14^. These gender-dependent differences are likely due to hormonal influence on erythropoiesis and menstrual blood loss in women ^16^^, ^^21^^-^^22^.

The analysis among the four age groups showed significant differences in the mean RBC values between age groups of 30-40 and >40, while significant differences in the mean MCH values were between age groups of <30 and 41-50. No significant age differences observed in other parameters.

The comparison of reference intervals, stratified by both gender and age, showed that there were significant age-related differences in the mean values of RBC, HGB, HCT, MCV and MCH among males. In other words, the distribution of RBC, HGB and HCT mean values almost showed an age-related decline in males, while positive correlation was almost found between age groups and both MCV and MCH mean values. Besides, it can be concluded that in both genders, mean values of PLT increased until the age of 50, but then declined. The decline in PLT after the age of 50 may reflect a reduction in hematopoietic stem cell reserve during aging or a survival advantage in the subjects with lower platelet counts^23^. Moreover, there were also significant age-related differences in the WBC mean values among females between age groups of <30 and 41-50. However, according to Figure1 and Table 2, there were some differences in RBC, HGB and HCT values by gender, indicating that males have higher values than females in all age groups. But MCH values of females in all age groups and WBC and PLT counts in females older than 30 years were higher compared to the males in the same age group. The above-mentioned results reinforce the need to establish age-specific reference intervals for both genders.

By comparing hematological reference Intervals, it was found that the lower limits of the hematological reference Intervals obtained in the current study were higher than those derived from the US population (Table 4). Besides, the differences between the lower and upper limits of hematological reference values were much less in this study than those in the US population.

The more narrow range may be attributed to selection of healthy blood donors in a specific age cohort as a sample. According to the results, All hematological mean values in the US population was included in the 95% confidence intervals for all those in our study, except HCT. 

Our study comes with several limitations: 1) the lack of detailed information to rule out subclinical conditions because we conducted this study within the framework of the blood bank’s existing procedures without additional staff or infrastructure and without resources spent on volunteer recruitment, 2) The calculation of ranges based on a very specific population (anonymous blood bank donors), 3) The disproportionate number of females to males (17% vs. 83%) due to less frequent participation of females in blood donation, 4) The insufficient number of female samples (Non-compliance with the recommended CLSI sample size of 120 per every partitioned group ^24^ ,5) the lack of age groups younger than 19 and older than 64 years . 

The power of study was that the participants in this study were voluntary blood donors from the general population and their health status was rigorously evaluated by clinical history, examination and laboratory tests. Therefore, the reference intervals obtained are likely to be representative of healthy Iranian adults. Besides, although few studies tried to show hematological profiles of Iranian population^20^^,^^25^, to the best of the authors’ knowledge, the present study is more comprehensive in terms of the quantity and type of hematological parameters included, and also in taking into account age-and gender-dependent differences among hematological values.

## CONCLUSION

 In conclusion, this study provides the gender and age-specific, locally derived hematological reference intervals of healthy Iranian blood donors for use in health care and clinical research. As age groups younger than 19 and older than 64 years were not included in this study, conducting similar nationwide study is recommended to determine the gender-and age-based hematological reference intervals of the Iranian population as a whole. 
